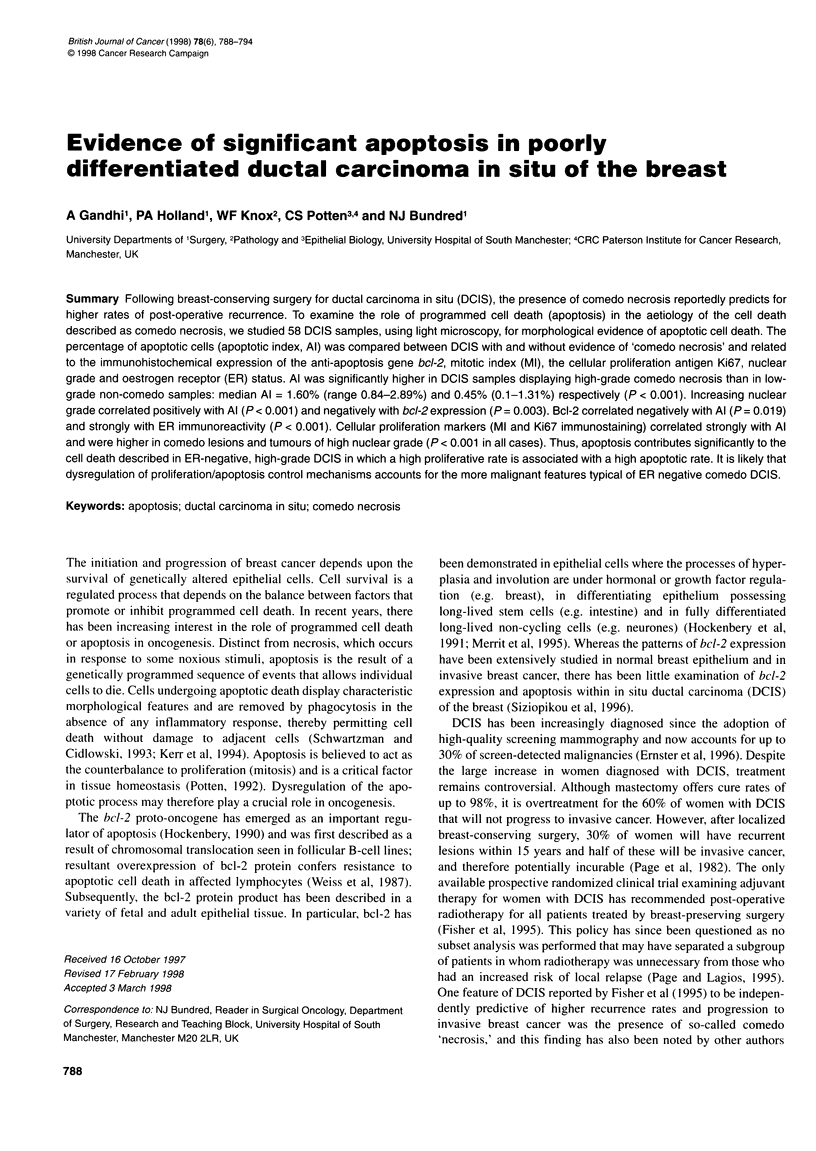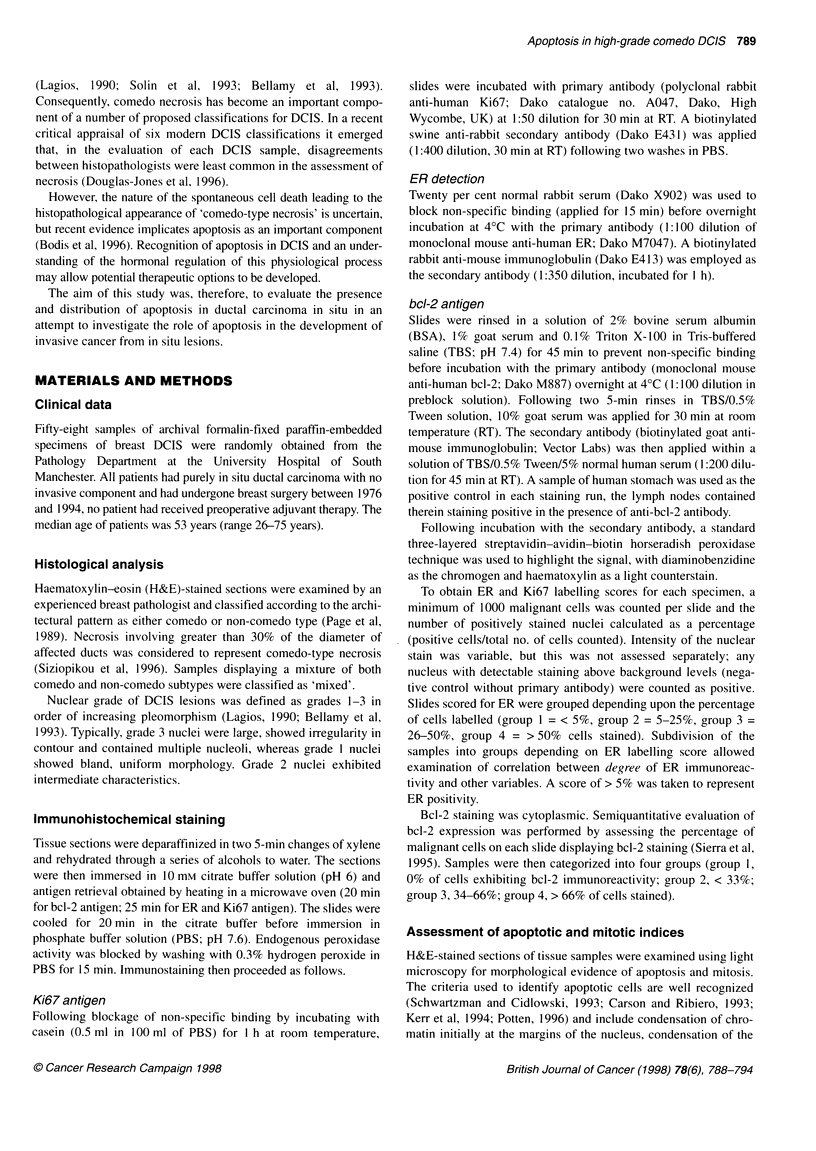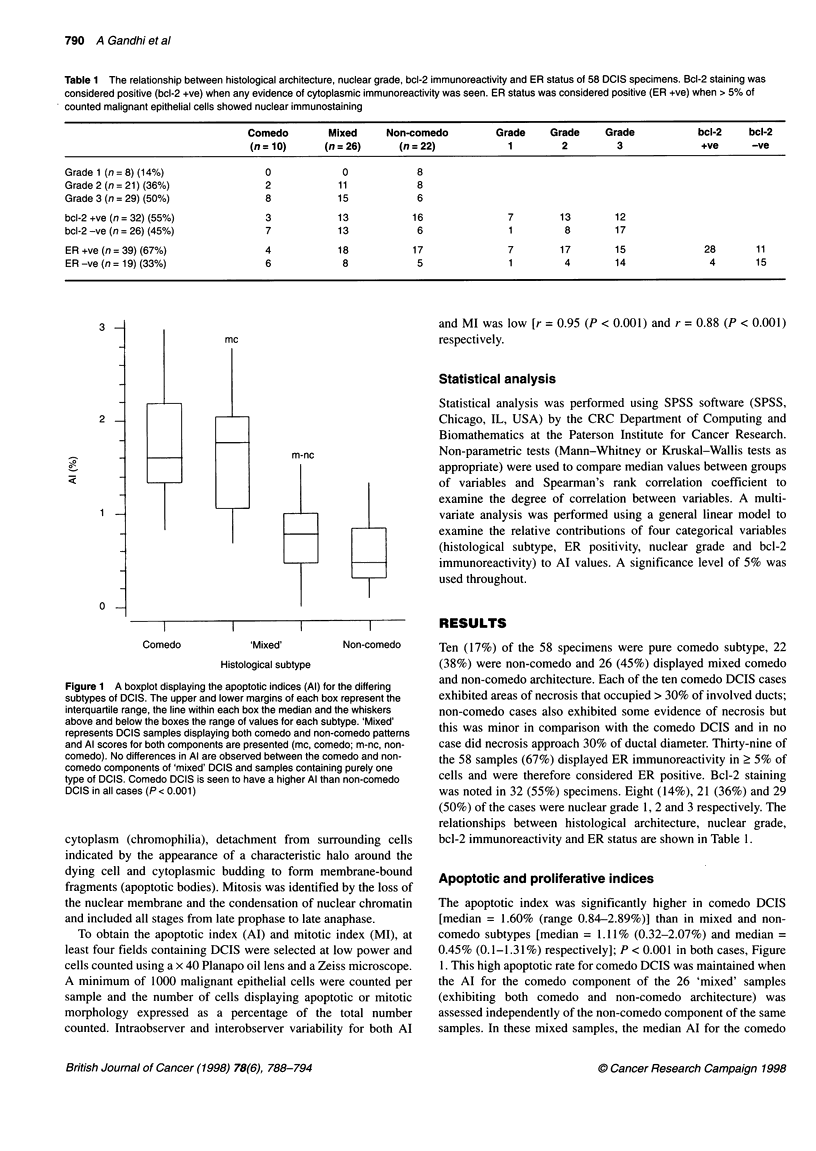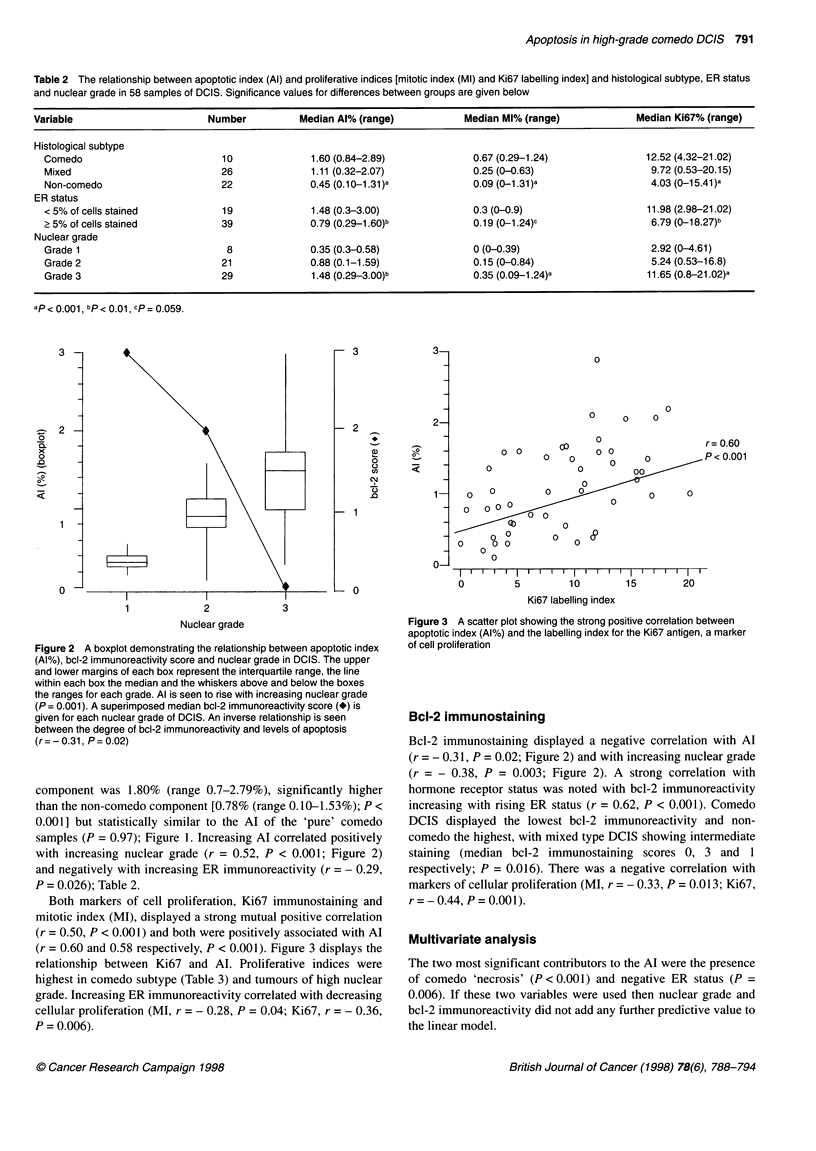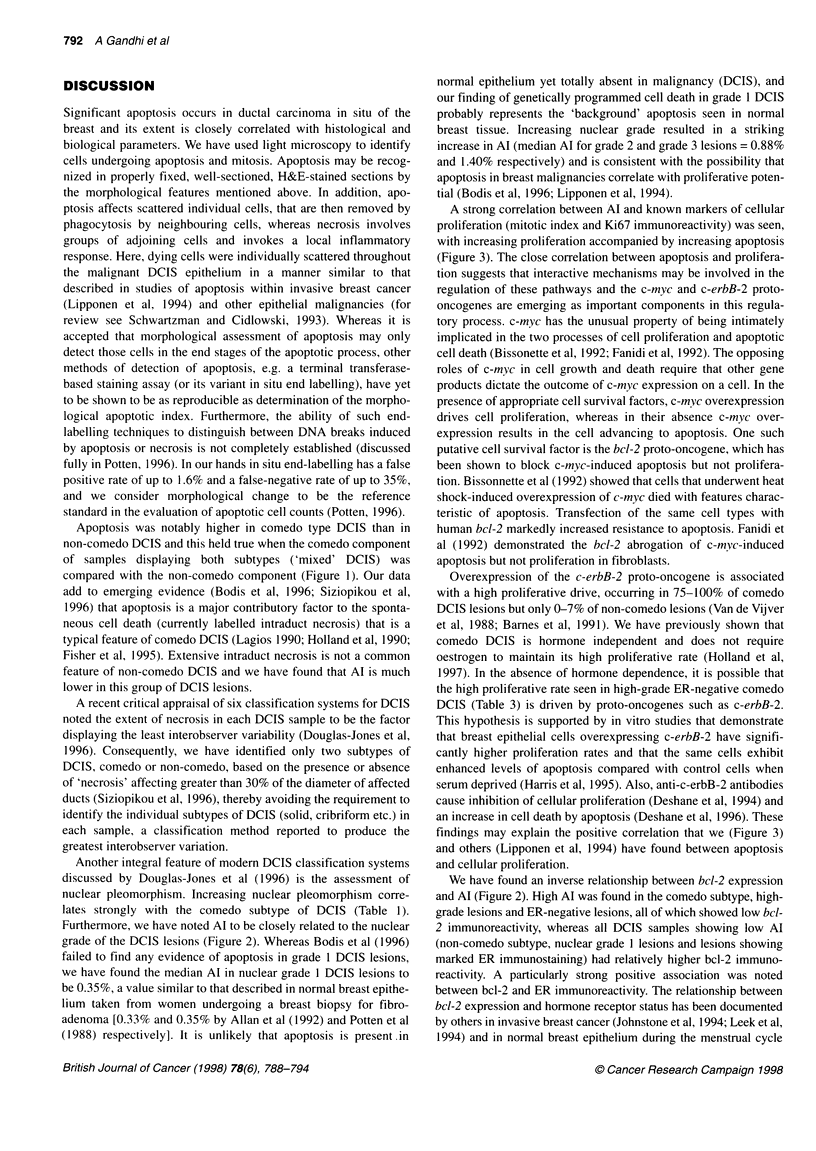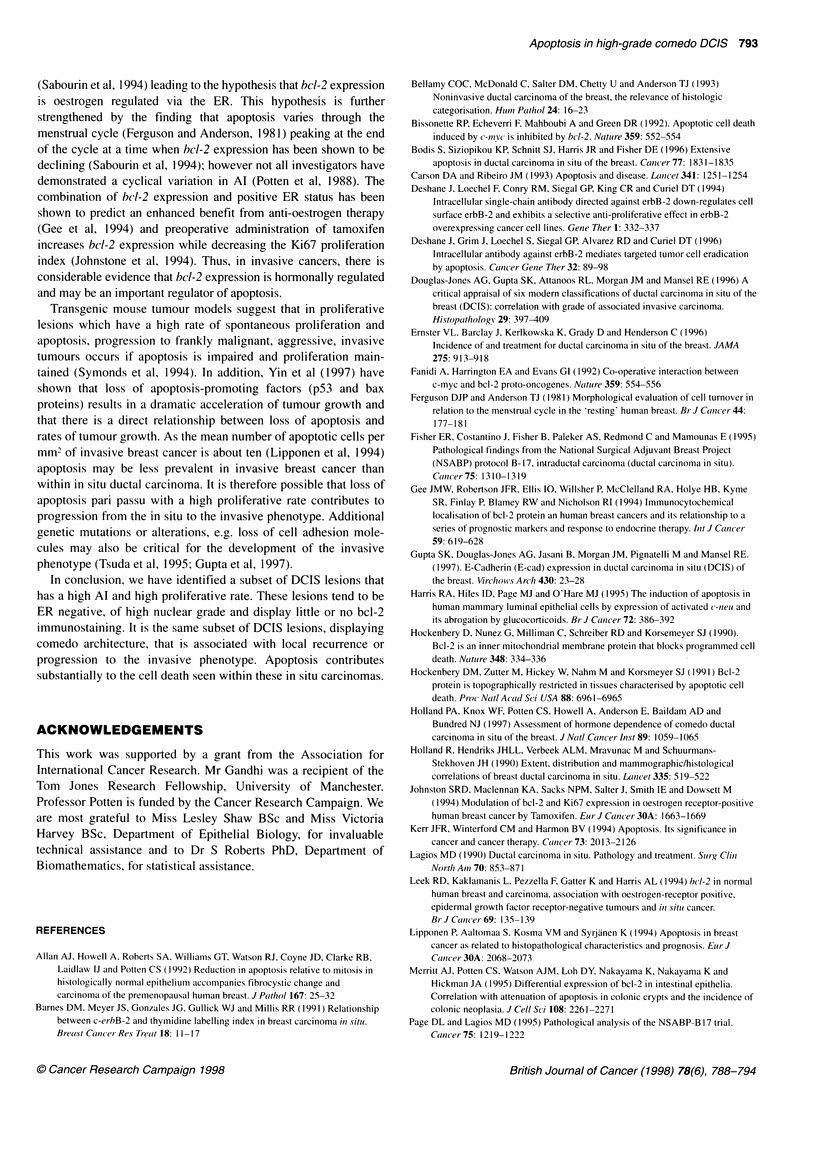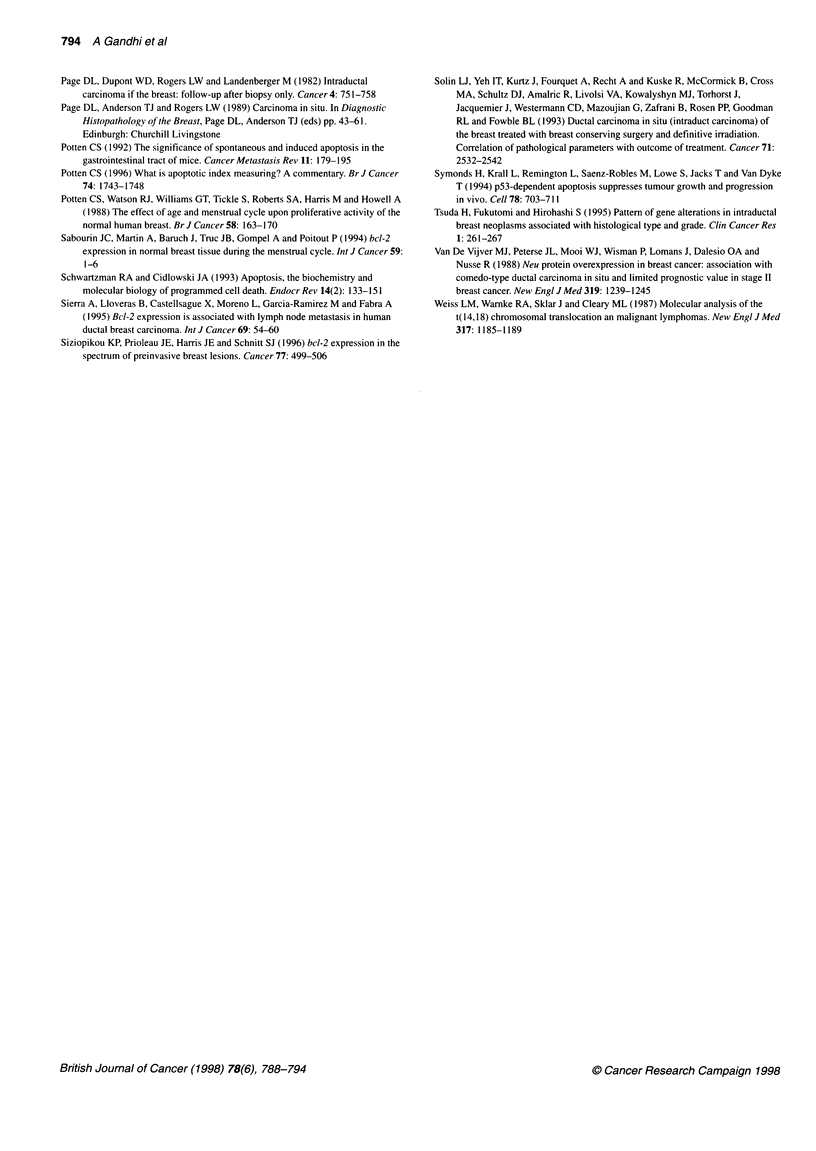# Evidence of significant apoptosis in poorly differentiated ductal carcinoma in situ of the breast.

**DOI:** 10.1038/bjc.1998.580

**Published:** 1998-09

**Authors:** A. Gandhi, P. A. Holland, W. F. Knox, C. S. Potten, N. J. Bundred

**Affiliations:** University Department of Surgery, University Hospital of South Manchester, UK.

## Abstract

Following breast-conserving surgery for ductal carcinoma in situ (DCIS), the presence of comedo necrosis reportedly predicts for higher rates of post-operative recurrence. To examine the role of programmed cell death (apoptosis) in the aetiology of the cell death described as comedo necrosis, we studied 58 DCIS samples, using light microscopy, for morphological evidence of apoptotic cell death. The percentage of apoptotic cells (apoptotic index, AI) was compared between DCIS with and without evidence of 'comedo necrosis' and related to the immunohistochemical expression of the anti-apoptosis gene bcl-2, mitotic index (MI), the cellular proliferation antigen Ki67, nuclear grade and oestrogen receptor (ER) status. AI was significantly higher in DCIS samples displaying high-grade comedo necrosis than in low-grade non-comedo samples: median AI = 1.60% (range 0.84-2.89%) and 0.45% (0.1-1.31%) respectively (P < 0.001). Increasing nuclear grade correlated positively with AI (P < 0.001) and negatively with bcl-2 expression (P = 0.003). Bcl-2 correlated negatively with AI (P = 0.019) and strongly with ER immunoreactivity (P < 0.001). Cellular proliferation markers (MI and Ki67 immunostaining) correlated strongly with AI and were higher in comedo lesions and tumours of high nuclear grade (P < 0.001 in all cases). Thus, apoptosis contributes significantly to the cell death described in ER-negative, high-grade DCIS in which a high proliferative rate is associated with a high apoptotic rate. It is likely that dysregulation of proliferation/apoptosis control mechanisms accounts for the more malignant features typical of ER negative comedo DCIS.


British Journal of Cancer (1998) 78(6), 788–794
© 1998 Cancer Research Campaign

### Evidence of significant apoptosis in poorly differentiated ductal carcinoma in situ of the breast

**A Gandhi[1], PA Holland[1], WF Knox[2], CS Potten[3,4] and NJ Bundred[1]**

University Departments of [1]Surgery, [2]Pathology and [3]Epithelial Biology, University Hospital of South Manchester; [4]CRC Paterson Institute for Cancer Research, Manchester, UK

**Summary** Following breast-conserving surgery for ductal carcinoma in situ (DCIS), the presence of comedo necrosis reportedly predicts for higher rates of post-operative recurrence. To examine the role of programmed cell death (apoptosis) in the aetiology of the cell death described as comedo necrosis, we studied 58 DCIS samples, using light microscopy, for morphological evidence of apoptotic cell death. The percentage of apoptotic cells (apoptotic index, AI) was compared between DCIS with and without evidence of 'comedo necrosis' and related to the immunohistochemical expression of the anti-apoptosis gene *bcl-2*, mitotic index (MI), the cellular proliferation antigen Ki67, nuclear grade and oestrogen receptor (ER) status. AI was significantly higher in DCIS samples displaying high-grade comedo necrosis than in low-grade non-comedo samples: median AI = 1.60% (range 0.84–2.89%) and 0.45% (0.1–1.31%) respectively ($P < 0.001$). Increasing nuclear grade correlated positively with AI ($P < 0.001$) and negatively with *bcl-2* expression ($P = 0.003$). Bcl-2 correlated negatively with AI ($P = 0.019$) and strongly with ER immunoreactivity ($P < 0.001$). Cellular proliferation markers (MI and Ki67 immunostaining) correlated strongly with AI and were higher in comedo lesions and tumours of high nuclear grade ($P < 0.001$ in all cases). Thus, apoptosis contributes significantly to the cell death described in ER-negative, high-grade DCIS in which a high proliferative rate is associated with a high apoptotic rate. It is likely that dysregulation of proliferation/apoptosis control mechanisms accounts for the more malignant features typical of ER negative comedo DCIS.

**Keywords:** apoptosis; ductal carcinoma in situ; comedo necrosis

The initiation and progression of breast cancer depends upon the survival of genetically altered epithelial cells. Cell survival is a regulated process that depends on the balance between factors that promote or inhibit programmed cell death. In recent years, there has been increasing interest in the role of programmed cell death or apoptosis in oncogenesis. Distinct from necrosis, which occurs in response to some noxious stimuli, apoptosis is the result of a genetically programmed sequence of events that allows individual cells to die. Cells undergoing apoptotic death display characteristic morphological features and are removed by phagocytosis in the absence of any inflammatory response, thereby permitting cell death without damage to adjacent cells (Schwartzman and Cidlowski, 1993; Kerr et al, 1994). Apoptosis is believed to act as the counterbalance to proliferation (mitosis) and is a critical factor in tissue homeostasis (Potten, 1992). Dysregulation of the apoptotic process may therefore play a crucial role in oncogenesis.

The *bcl-2* proto-oncogene has emerged as an important regulator of apoptosis (Hockenbery, 1990) and was first described as a result of chromosomal translocation seen in follicular B-cell lines; resultant overexpression of bcl-2 protein confers resistance to apoptotic cell death in affected lymphocytes (Weiss et al, 1987). Subsequently, the bcl-2 protein product has been described in a variety of fetal and adult epithelial tissue. In particular, bcl-2 has been demonstrated in epithelial cells where the processes of hyperplasia and involution are under hormonal or growth factor regulation (e.g. breast), in differentiating epithelium possessing long-lived stem cells (e.g. intestine) and in fully differentiated long-lived non-cycling cells (e.g. neurones) (Hockenbery et al, 1991; Merrit et al, 1995). Whereas the patterns of *bcl-2* expression have been extensively studied in normal breast epithelium and in invasive breast cancer, there has been little examination of *bcl-2* expression and apoptosis within in situ ductal carcinoma (DCIS) of the breast (Siziopikou et al, 1996).

DCIS has been increasingly diagnosed since the adoption of high-quality screening mammography and now accounts for up to 30% of screen-detected malignancies (Ernster et al, 1996). Despite the large increase in women diagnosed with DCIS, treatment remains controversial. Although mastectomy offers cure rates of up to 98%, it is overtreatment for the 60% of women with DCIS that will not progress to invasive cancer. However, after localized breast-conserving surgery, 30% of women will have recurrent lesions within 15 years and half of these will be invasive cancer, and therefore potentially incurable (Page et al, 1982). The only available prospective randomized clinical trial examining adjuvant therapy for women with DCIS has recommended post-operative radiotherapy for all patients treated by breast-preserving surgery (Fisher et al, 1995). This policy has since been questioned as no subset analysis was performed that may have separated a subgroup of patients in whom radiotherapy was unnecessary from those who had an increased risk of local relapse (Page and Lagios, 1995). One feature of DCIS reported by Fisher et al (1995) to be independently predictive of higher recurrence rates and progression to invasive breast cancer was the presence of so-called comedo 'necrosis,' and this finding has also been noted by other authors

*Received 16 October 1997*
*Revised 17 February 1998*
*Accepted 3 March 1998*

*Correspondence to:* NJ Bundred, Reader in Surgical Oncology, Department of Surgery, Research and Teaching Block, University Hospital of South Manchester, Manchester M20 2LR, UK

(Lagios, 1990; Solin et al, 1993; Bellamy et al, 1993). Consequently, comedo necrosis has become an important component of a number of proposed classifications for DCIS. In a recent critical appraisal of six modern DCIS classifications it emerged that, in the evaluation of each DCIS sample, disagreements between histopathologists were least common in the assessment of necrosis (Douglas-Jones et al, 1996).

However, the nature of the spontaneous cell death leading to the histopathological appearance of 'comedo-type necrosis' is uncertain, but recent evidence implicates apoptosis as an important component (Bodis et al, 1996). Recognition of apoptosis in DCIS and an understanding of the hormonal regulation of this physiological process may allow potential therapeutic options to be developed.

The aim of this study was, therefore, to evaluate the presence and distribution of apoptosis in ductal carcinoma in situ in an attempt to investigate the role of apoptosis in the development of invasive cancer from in situ lesions.

### MATERIALS AND METHODS

### Clinical data

Fifty-eight samples of archival formalin-fixed paraffin-embedded specimens of breast DCIS were randomly obtained from the Pathology Department at the University Hospital of South Manchester. All patients had purely in situ ductal carcinoma with no invasive component and had undergone breast surgery between 1976 and 1994, no patient had received preoperative adjuvant therapy. The median age of patients was 53 years (range 26–75 years).

### Histological analysis

Haematoxylin–eosin (H&E)-stained sections were examined by an experienced breast pathologist and classified according to the architectural pattern as either comedo or non-comedo type (Page et al, 1989). Necrosis involving greater than 30% of the diameter of affected ducts was considered to represent comedo-type necrosis (Siziopikou et al, 1996). Samples displaying a mixture of both comedo and non-comedo subtypes were classified as 'mixed'.

Nuclear grade of DCIS lesions was defined as grades 1–3 in order of increasing pleomorphism (Lagios, 1990; Bellamy et al, 1993). Typically, grade 3 nuclei were large, showed irregularity in contour and contained multiple nucleoli, whereas grade 1 nuclei showed bland, uniform morphology. Grade 2 nuclei exhibited intermediate characteristics.

### Immunohistochemical staining

Tissue sections were deparaffinized in two 5-min changes of xylene and rehydrated through a series of alcohols to water. The sections were then immersed in 10 mM citrate buffer solution (pH 6) and antigen retrieval obtained by heating in a microwave oven (20 min for bcl-2 antigen; 25 min for ER and Ki67 antigen). The slides were cooled for 20 min in the citrate buffer before immersion in phosphate buffer solution (PBS; pH 7.6). Endogenous peroxidase activity was blocked by washing with 0.3% hydrogen peroxide in PBS for 15 min. Immunostaining then proceeded as follows.

#### *Ki67 antigen*

Following blockage of non-specific binding by incubating with casein (0.5 ml in 100 ml of PBS) for 1 h at room temperature, slides were incubated with primary antibody (polyclonal rabbit anti-human Ki67; Dako catalogue no. A047, Dako, High Wycombe, UK) at 1:50 dilution for 30 min at RT. A biotinylated swine anti-rabbit secondary antibody (Dako E431) was applied (1:400 dilution, 30 min at RT) following two washes in PBS.

#### *ER detection*

Twenty per cent normal rabbit serum (Dako X902) was used to block non-specific binding (applied for 15 min) before overnight incubation at 4°C with the primary antibody (1:100 dilution of monoclonal mouse anti-human ER; Dako M7047). A biotinylated rabbit anti-mouse immunoglobulin (Dako E413) was employed as the secondary antibody (1:350 dilution, incubated for 1 h).

#### *bcl-2 antigen*

Slides were rinsed in a solution of 2% bovine serum albumin (BSA), 1% goat serum and 0.1% Triton X-100 in Tris-buffered saline (TBS; pH 7.4) for 45 min to prevent non-specific binding before incubation with the primary antibody (monoclonal mouse anti-human bcl-2; Dako M887) overnight at 4°C (1:100 dilution in preblock solution). Following two 5-min rinses in TBS/0.5% Tween solution, 10% goat serum was applied for 30 min at room temperature (RT). The secondary antibody (biotinylated goat anti-mouse immunoglobulin; Vector Labs) was then applied within a solution of TBS/0.5% Tween/5% normal human serum (1:200 dilution for 45 min at RT). A sample of human stomach was used as the positive control in each staining run, the lymph nodes contained therein staining positive in the presence of anti-bcl-2 antibody.

Following incubation with the secondary antibody, a standard three-layered streptavidin–avidin–biotin horseradish peroxidase technique was used to highlight the signal, with diaminobenzidine as the chromogen and haematoxylin as a light counterstain.

To obtain ER and Ki67 labelling scores for each specimen, a minimum of 1000 malignant cells was counted per slide and the number of positively stained nuclei calculated as a percentage (positive cells/total no. of cells counted). Intensity of the nuclear stain was variable, but this was not assessed separately; any nucleus with detectable staining above background levels (negative control without primary antibody) were counted as positive. Slides scored for ER were grouped depending upon the percentage of cells labelled (group 1 = < 5%, group 2 = 5–25%, group 3 = 26–50%, group 4 = > 50% cells stained). Subdivision of the samples into groups depending on ER labelling score allowed examination of correlation between *degree* of ER immunoreactivity and other variables. A score of > 5% was taken to represent ER positivity.

Bcl-2 staining was cytoplasmic. Semiquantitative evaluation of bcl-2 expression was performed by assessing the percentage of malignant cells on each slide displaying bcl-2 staining (Sierra et al, 1995). Samples were then categorized into four groups (group 1, 0% of cells exhibiting bcl-2 immunoreactivity; group 2, < 33%; group 3, 34–66%; group 4, > 66% of cells stained).

### Assessment of apoptotic and mitotic indices

H&E-stained sections of tissue samples were examined using light microscopy for morphological evidence of apoptosis and mitosis. The criteria used to identify apoptotic cells are well recognized (Schwartzman and Cidlowski, 1993; Carson and Ribiero, 1993; Kerr et al, 1994; Potten, 1996) and include condensation of chromatin initially at the margins of the nucleus, condensation of the

© Cancer Research Campaign 1998

**Table 1** The relationship between histological architecture, nuclear grade, bcl-2 immunoreactivity and ER status of 58 DCIS specimens. Bcl-2 staining was considered positive (bcl-2 +ve) when any evidence of cytoplasmic immunoreactivity was seen. ER status was considered positive (ER +ve) when > 5% of counted malignant epithelial cells showed nuclear immunostaining

| | Comedo (*n* = 10) | Mixed (*n* = 26) | Non-comedo (*n* = 22) | Grade 1 | Grade 2 | Grade 3 | bcl-2 +ve | bcl-2 –ve |
|---|---|---|---|---|---|---|---|---|
| Grade 1 (*n* = 8) (14%) | 0 | 0 | 8 | | | | | |
| Grade 2 (*n* = 21) (36%) | 2 | 11 | 8 | | | | | |
| Grade 3 (*n* = 29) (50%) | 8 | 15 | 6 | | | | | |
| bcl-2 +ve (*n* = 32) (55%) | 3 | 13 | 16 | 7 | 13 | 12 | | |
| bcl-2 –ve (*n* = 26) (45%) | 7 | 13 | 6 | 1 | 8 | 17 | | |
| ER +ve (*n* = 39) (67%) | 4 | 18 | 17 | 7 | 17 | 15 | 28 | 11 |
| ER –ve (*n* = 19) (33%) | 6 | 8 | 5 | 1 | 4 | 14 | 4 | 15 |

**Figure 1** A boxplot displaying the apoptotic indices (AI) for the differing subtypes of DCIS. The upper and lower margins of each box represent the interquartile range, the line within each box the median and the whiskers above and below the boxes the range of values for each subtype. 'Mixed' represents DCIS samples displaying both comedo and non-comedo patterns and AI scores for both components are presented (mc, comedo; m-nc, non-comedo). No differences in AI are observed between the comedo and non-comedo components of 'mixed' DCIS and samples containing purely one type of DCIS. Comedo DCIS is seen to have a higher AI than non-comedo DCIS in all cases ($P < 0.001$)

cytoplasm (chromophilia), detachment from surrounding cells indicated by the appearance of a characteristic halo around the dying cell and cytoplasmic budding to form membrane-bound fragments (apoptotic bodies). Mitosis was identified by the loss of the nuclear membrane and the condensation of nuclear chromatin and included all stages from late prophase to late anaphase.

To obtain the apoptotic index (AI) and mitotic index (MI), at least four fields containing DCIS were selected at low power and cells counted using a × 40 Planapo oil lens and a Zeiss microscope. A minimum of 1000 malignant epithelial cells were counted per sample and the number of cells displaying apoptotic or mitotic morphology expressed as a percentage of the total number counted. Intraobserver and interobserver variability for both AI and MI was low [$r = 0.95$ ($P < 0.001$) and $r = 0.88$ ($P < 0.001$) respectively.

### Statistical analysis

Statistical analysis was performed using SPSS software (SPSS, Chicago, IL, USA) by the CRC Department of Computing and Biomathematics at the Paterson Institute for Cancer Research. Non-parametric tests (Mann–Whitney or Kruskal–Wallis tests as appropriate) were used to compare median values between groups of variables and Spearman's rank correlation coefficient to examine the degree of correlation between variables. A multivariate analysis was performed using a general linear model to examine the relative contributions of four categorical variables (histological subtype, ER positivity, nuclear grade and bcl-2 immunoreactivity) to AI values. A significance level of 5% was used throughout.

### RESULTS

Ten (17%) of the 58 specimens were pure comedo subtype, 22 (38%) were non-comedo and 26 (45%) displayed mixed comedo and non-comedo architecture. Each of the ten comedo DCIS cases exhibited areas of necrosis that occupied > 30% of involved ducts; non-comedo cases also exhibited some evidence of necrosis but this was minor in comparison with the comedo DCIS and in no case did necrosis approach 30% of ductal diameter. Thirty-nine of the 58 samples (67%) displayed ER immunoreactivity in ≥ 5% of cells and were therefore considered ER positive. Bcl-2 staining was noted in 32 (55%) specimens. Eight (14%), 21 (36%) and 29 (50%) of the cases were nuclear grade 1, 2 and 3 respectively. The relationships between histological architecture, nuclear grade, bcl-2 immunoreactivity and ER status are shown in Table 1.

### Apoptotic and proliferative indices

The apoptotic index was significantly higher in comedo DCIS [median = 1.60% (range 0.84–2.89%)] than in mixed and non-comedo subtypes [median = 1.11% (0.32–2.07%) and median = 0.45% (0.1–1.31%) respectively]; $P < 0.001$ in both cases, Figure 1. This high apoptotic rate for comedo DCIS was maintained when the AI for the comedo component of the 26 'mixed' samples (exhibiting both comedo and non-comedo architecture) was assessed independently of the non-comedo component of the same samples. In these mixed samples, the median AI for the comedo

© Cancer Research Campaign 1998

**Table 2** The relationship between apoptotic index (AI) and proliferative indices [mitotic index (MI) and Ki67 labelling index] and histological subtype, ER status and nuclear grade in 58 samples of DCIS. Significance values for differences between groups are given below

| Variable | Number | Median AI% (range) | Median MI% (range) | Median Ki67% (range) |
|---|---|---|---|---|
| Histological subtype | | | | |
| Comedo | 10 | 1.60 (0.84–2.89) | 0.67 (0.29–1.24) | 12.52 (4.32–21.02) |
| Mixed | 26 | 1.11 (0.32–2.07) | 0.25 (0–0.63) | 9.72 (0.53–20.15) |
| Non-comedo | 22 | 0.45 (0.10–1.31)[a] | 0.09 (0–1.31)[a] | 4.03 (0–15.41)[a] |
| ER status | | | | |
| < 5% of cells stained | 19 | 1.48 (0.3–3.00) | 0.3 (0–0.9) | 11.98 (2.98–21.02) |
| ≥ 5% of cells stained | 39 | 0.79 (0.29–1.60)[b] | 0.19 (0–1.24)[c] | 6.79 (0–18.27)[b] |
| Nuclear grade | | | | |
| Grade 1 | 8 | 0.35 (0.3–0.58) | 0 (0–0.39) | 2.92 (0–4.61) |
| Grade 2 | 21 | 0.88 (0.1–1.59) | 0.15 (0–0.84) | 5.24 (0.53–16.8) |
| Grade 3 | 29 | 1.48 (0.29–3.00)[b] | 0.35 (0.09–1.24)[a] | 11.65 (0.8–21.02)[a] |

[a]$P < 0.001$, [b]$P < 0.01$, [c]$P = 0.059$.

**Figure 2** A boxplot demonstrating the relationship between apoptotic index (AI%), bcl-2 immunoreactivity score and nuclear grade in DCIS. The upper and lower margins of each box represent the interquartile range, the line within each box the median and the whiskers above and below the boxes the ranges for each grade. AI is seen to rise with increasing nuclear grade ($P = 0.001$). A superimposed median bcl-2 immunoreactivity score (◆) is given for each nuclear grade of DCIS. An inverse relationship is seen between the degree of bcl-2 immunoreactivity and levels of apoptosis ($r = -0.31$, $P = 0.02$)

**Figure 3** A scatter plot showing the strong positive correlation between apoptotic index (AI%) and the labelling index for the Ki67 antigen, a marker of cell proliferation component was 1.80% (range 0.7–2.79%), significantly higher than the non-comedo component [0.78% (range 0.10–1.53%); $P < 0.001$] but statistically similar to the AI of the 'pure' comedo samples ($P = 0.97$); Figure 1. Increasing AI correlated positively with increasing nuclear grade ($r = 0.52$, $P < 0.001$; Figure 2) and negatively with increasing ER immunoreactivity ($r = -0.29$, $P = 0.026$); Table 2.

Both markers of cell proliferation, Ki67 immunostaining and mitotic index (MI), displayed a strong mutual positive correlation ($r = 0.50$, $P < 0.001$) and both were positively associated with AI ($r = 0.60$ and 0.58 respectively, $P < 0.001$). Figure 3 displays the relationship between Ki67 and AI. Proliferative indices were highest in comedo subtype (Table 3) and tumours of high nuclear grade. Increasing ER immunoreactivity correlated with decreasing cellular proliferation (MI, $r = -0.28$, $P = 0.04$; Ki67, $r = -0.36$, $P = 0.006$).

### Bcl-2 immunostaining

Bcl-2 immunostaining displayed a negative correlation with AI ($r = -0.31$, $P = 0.02$; Figure 2) and with increasing nuclear grade ($r = -0.38$, $P = 0.003$; Figure 2). A strong correlation with hormone receptor status was noted with bcl-2 immunoreactivity increasing with rising ER status ($r = 0.62$, $P < 0.001$). Comedo DCIS displayed the lowest bcl-2 immunoreactivity and non-comedo the highest, with mixed type DCIS showing intermediate staining (median bcl-2 immunostaining scores 0, 3 and 1 respectively; $P = 0.016$). There was a negative correlation with markers of cellular proliferation (MI, $r = -0.33$, $P = 0.013$; Ki67, $r = -0.44$, $P = 0.001$).

### Multivariate analysis

The two most significant contributors to the AI were the presence of comedo 'necrosis' ($P < 0.001$) and negative ER status ($P = 0.006$). If these two variables were used then nuclear grade and bcl-2 immunoreactivity did not add any further predictive value to the linear model.

© Cancer Research Campaign 1998

### DISCUSSION

Significant apoptosis occurs in ductal carcinoma in situ of the breast and its extent is closely correlated with histological and biological parameters. We have used light microscopy to identify cells undergoing apoptosis and mitosis. Apoptosis may be recognized in properly fixed, well-sectioned, H&E-stained sections by the morphological features mentioned above. In addition, apoptosis affects scattered individual cells, that are then removed by phagocytosis by neighbouring cells, whereas necrosis involves groups of adjoining cells and invokes a local inflammatory response. Here, dying cells were individually scattered throughout the malignant DCIS epithelium in a manner similar to that described in studies of apoptosis within invasive breast cancer (Lipponen et al, 1994) and other epithelial malignancies (for review see Schwartzman and Cidlowski, 1993). Whereas it is accepted that morphological assessment of apoptosis may only detect those cells in the end stages of the apoptotic process, other methods of detection of apoptosis, e.g. a terminal transferase-based staining assay (or its variant in situ end labelling), have yet to be shown to be as reproducible as determination of the morphological apoptotic index. Furthermore, the ability of such end-labelling techniques to distinguish between DNA breaks induced by apoptosis or necrosis is not completely established (discussed fully in Potten, 1996). In our hands in situ end-labelling has a false positive rate of up to 1.6% and a false-negative rate of up to 35%, and we consider morphological change to be the reference standard in the evaluation of apoptotic cell counts (Potten, 1996).

Apoptosis was notably higher in comedo type DCIS than in non-comedo DCIS and this held true when the comedo component of samples displaying both subtypes ('mixed' DCIS) was compared with the non-comedo component (Figure 1). Our data add to emerging evidence (Bodis et al, 1996; Siziopikou et al, 1996) that apoptosis is a major contributory factor to the spontaneous cell death (currently labelled intraduct necrosis) that is a typical feature of comedo DCIS (Lagios 1990; Holland et al, 1990; Fisher et al, 1995). Extensive intraduct necrosis is not a common feature of non-comedo DCIS and we have found that AI is much lower in this group of DCIS lesions.

A recent critical appraisal of six classification systems for DCIS noted the extent of necrosis in each DCIS sample to be the factor displaying the least interobserver variability (Douglas-Jones et al, 1996). Consequently, we have identified only two subtypes of DCIS, comedo or non-comedo, based on the presence or absence of 'necrosis' affecting greater than 30% of the diameter of affected ducts (Siziopikou et al, 1996), thereby avoiding the requirement to identify the individual subtypes of DCIS (solid, cribriform etc.) in each sample, a classification method reported to produce the greatest interobserver variation.

Another integral feature of modern DCIS classification systems discussed by Douglas-Jones et al (1996) is the assessment of nuclear pleomorphism. Increasing nuclear pleomorphism correlates strongly with the comedo subtype of DCIS (Table 1). Furthermore, we have noted AI to be closely related to the nuclear grade of the DCIS lesions (Figure 2). Whereas Bodis et al (1996) failed to find any evidence of apoptosis in grade 1 DCIS lesions, we have found the median AI in nuclear grade 1 DCIS lesions to be 0.35%, a value similar to that described in normal breast epithelium taken from women undergoing a breast biopsy for fibroadenoma [0.33% and 0.35% by Allan et al (1992) and Potten et al (1988) respectively]. It is unlikely that apoptosis is present in normal epithelium yet totally absent in malignancy (DCIS), and our finding of genetically programmed cell death in grade 1 DCIS probably represents the 'background' apoptosis seen in normal breast tissue. Increasing nuclear grade resulted in a striking increase in AI (median AI for grade 2 and grade 3 lesions = 0.88% and 1.40% respectively) and is consistent with the possibility that apoptosis in breast malignancies correlate with proliferative potential (Bodis et al, 1996; Lipponen et al, 1994).

A strong correlation between AI and known markers of cellular proliferation (mitotic index and Ki67 immunoreactivity) was seen, with increasing proliferation accompanied by increasing apoptosis (Figure 3). The close correlation between apoptosis and proliferation suggests that interactive mechanisms may be involved in the regulation of these pathways and the c-*myc* and c-*erbB*-2 proto-oncogenes are emerging as important components in this regulatory process. c-*myc* has the unusual property of being intimately implicated in the two processes of cell proliferation and apoptotic cell death (Bissonette et al, 1992; Fanidi et al, 1992). The opposing roles of c-*myc* in cell growth and death require that other gene products dictate the outcome of c-*myc* expression on a cell. In the presence of appropriate cell survival factors, c-*myc* overexpression drives cell proliferation, whereas in their absence c-*myc* overexpression results in the cell advancing to apoptosis. One such putative cell survival factor is the *bcl-2* proto-oncogene, which has been shown to block c-*myc*-induced apoptosis but not proliferation. Bissonnette et al (1992) showed that cells that underwent heat shock-induced overexpression of *c-myc* died with features characteristic of apoptosis. Transfection of the same cell types with human *bcl-2* markedly increased resistance to apoptosis. Fanidi et al (1992) demonstrated the *bcl-2* abrogation of c-*myc*-induced apoptosis but not proliferation in fibroblasts.

Overexpression of the *c-erbB-2* proto-oncogene is associated with a high proliferative drive, occurring in 75–100% of comedo DCIS lesions but only 0–7% of non-comedo lesions (Van de Vijver et al, 1988; Barnes et al, 1991). We have previously shown that comedo DCIS is hormone independent and does not require oestrogen to maintain its high proliferative rate (Holland et al, 1997). In the absence of hormone dependence, it is possible that the high proliferative rate seen in high-grade ER-negative comedo DCIS (Table 3) is driven by proto-oncogenes such as c-*erbB*-2. This hypothesis is supported by in vitro studies that demonstrate that breast epithelial cells overexpressing c-*erbB*-2 have significantly higher proliferation rates and that the same cells exhibit enhanced levels of apoptosis compared with control cells when serum deprived (Harris et al, 1995). Also, anti-c-erbB-2 antibodies cause inhibition of cellular proliferation (Deshane et al, 1994) and an increase in cell death by apoptosis (Deshane et al, 1996). These findings may explain the positive correlation that we (Figure 3) and others (Lipponen et al, 1994) have found between apoptosis and cellular proliferation.

We have found an inverse relationship between *bcl-2* expression and AI (Figure 2). High AI was found in the comedo subtype, high-grade lesions and ER-negative lesions, all of which showed low *bcl-2* immunoreactivity, whereas all DCIS samples showing low AI (non-comedo subtype, nuclear grade 1 lesions and lesions showing marked ER immunostaining) had relatively higher bcl-2 immunoreactivity. A particularly strong positive association was noted between bcl-2 and ER immunoreactivity. The relationship between *bcl-2* expression and hormone receptor status has been documented by others in invasive breast cancer (Johnstone et al, 1994; Leek et al, 1994) and in normal breast epithelium during the menstrual cycle

© Cancer Research Campaign 1998

(Sabourin et al, 1994) leading to the hypothesis that *bcl-2* expression is oestrogen regulated via the ER. This hypothesis is further strengthened by the finding that apoptosis varies through the menstrual cycle (Ferguson and Anderson, 1981) peaking at the end of the cycle at a time when *bcl-2* expression has been shown to be declining (Sabourin et al, 1994); however not all investigators have demonstrated a cyclical variation in AI (Potten et al, 1988). The combination of *bcl-2* expression and positive ER status has been shown to predict an enhanced benefit from anti-oestrogen therapy (Gee et al, 1994) and preoperative administration of tamoxifen increases *bcl-2* expression while decreasing the Ki67 proliferation index (Johnstone et al, 1994). Thus, in invasive cancers, there is considerable evidence that *bcl-2* expression is hormonally regulated and may be an important regulator of apoptosis.

Transgenic mouse tumour models suggest that in proliferative lesions which have a high rate of spontaneous proliferation and apoptosis, progression to frankly malignant, aggressive, invasive tumours occurs if apoptosis is impaired and proliferation maintained (Symonds et al, 1994). In addition, Yin et al (1997) have shown that loss of apoptosis-promoting factors (p53 and bax proteins) results in a dramatic acceleration of tumour growth and that there is a direct relationship between loss of apoptosis and rates of tumour growth. As the mean number of apoptotic cells per $mm^2$ of invasive breast cancer is about ten (Lipponen et al, 1994) apoptosis may be less prevalent in invasive breast cancer than within in situ ductal carcinoma. It is therefore possible that loss of apoptosis pari passu with a high proliferative rate contributes to progression from the in situ to the invasive phenotype. Additional genetic mutations or alterations, e.g. loss of cell adhesion molecules may also be critical for the development of the invasive phenotype (Tsuda et al, 1995; Gupta et al, 1997).

In conclusion, we have identified a subset of DCIS lesions that has a high AI and high proliferative rate. These lesions tend to be ER negative, of high nuclear grade and display little or no bcl-2 immunostaining. It is the same subset of DCIS lesions, displaying comedo architecture, that is associated with local recurrence or progression to the invasive phenotype. Apoptosis contributes substantially to the cell death seen within these in situ carcinomas.

### ACKNOWLEDGEMENTS

This work was supported by a grant from the Association for International Cancer Research. Mr Gandhi was a recipient of the Tom Jones Research Fellowship, University of Manchester. Professor Potten is funded by the Cancer Research Campaign. We are most grateful to Miss Lesley Shaw BSc and Miss Victoria Harvey BSc, Department of Epithelial Biology, for invaluable technical assistance and to Dr S Roberts PhD, Department of Biomathematics, for statistical assistance.

### REFERENCES

Allan AJ, Howell A, Roberts SA, Williams GT, Watson RJ, Coyne JD, Clarke RB, Laidlaw IJ and Potten CS (1992) Reduction in apoptosis relative to mitosis in histologically normal epithelium accompanies fibrocystic change and carcinoma of the premenopausal human breast. *J Pathol* **167**: 25–32

Barnes DM, Meyer JS, Gonzales JG, Gullick WJ and Millis RR (1991) Relationship between c-*erb*B-2 and thymidine labelling index in breast carcinoma *in situ*. *Breast Cancer Res Treat* **18**: 11–17

Bellamy COC, McDonald C, Salter DM, Chetty U and Anderson TJ (1993) Noninvasive ductal carcinoma of the breast, the relevance of histologic categorisation. *Hum Pathol* **24**: 16–23

Bissonette RP, Echeverri F, Mahboubi A and Green DR (1992). Apoptotic cell death induced by *c-myc* is inhibited by *bcl-2*. *Nature* **359**: 552–554

Bodis S, Siziopikou KP, Schnitt SJ, Harris JR and Fisher DE (1996) Extensive apoptosis in ductal carcinoma in situ of the breast. *Cancer* **77**: 1831–1835

Carson DA and Ribeiro JM (1993) Apoptosis and disease. *Lancet* **341**: 1251–1254

Deshane J, Loechel F, Conry RM, Siegal GP, King CR and Curiel DT (1994) Intracellular single-chain antibody directed against erbB-2 down-regulates cell surface erbB-2 and exhibits a selective anti-proliferative effect in erbB-2 overexpressing cancer cell lines. *Gene Ther* **1**: 332–337

Deshane J, Grim J, Loechel S, Siegal GP, Alvarez RD and Curiel DT (1996) Intracellular antibody against erbB-2 mediates targeted tumor cell eradication by apoptosis. *Cancer Gene Ther* **32**: 89–98

Douglas-Jones AG, Gupta SK, Attanoos RL, Morgan JM and Mansel RE (1996) A critical appraisal of six modern classifications of ductal carcinoma in situ of the breast (DCIS): correlation with grade of associated invasive carcinoma. *Histopathology* **29**: 397–409

Ernster VL, Barclay J, Kerlkowska K, Grady D and Henderson C (1996) Incidence of and treatment for ductal carcinoma in situ of the breast. *JAMA* **275**: 913–918

Fanidi A, Harrington EA and Evans GI (1992) Co-operative interaction between c-myc and bcl-2 proto-oncogenes. *Nature* **359**: 554–556

Ferguson DJP and Anderson TJ (1981) Morphological evaluation of cell turnover in relation to the menstrual cycle in the 'resting' human breast. *Br J Cancer* **44**: 177–181

Fisher ER, Costantino J, Fisher B, Paleker AS, Redmond C and Mamounas E (1995) Pathological findings from the National Surgical Adjuvant Breast Project (NSABP) protocol B-17, intraductal carcinoma (ductal carcinoma in situ). *Cancer* **75**: 1310–1319

Gee JMW, Robertson JFR, Ellis IO, Willsher P, McClelland RA, Holye HB, Kyme SR, Finlay P, Blamey RW and Nicholson RI (1994) Immunocytochemical localisation of bcl-2 protein an human breast cancers and its relationship to a series of prognostic markers and response to endocrine therapy. *Int J Cancer* **59**: 619–628

Gupta SK, Douglas-Jones AG, Jasani B, Morgan JM, Pignatelli M and Mansel RE. (1997). E-Cadherin (E-cad) expression in ductal carcinoma in situ (DCIS) of the breast. *Virchows Arch* **430**: 23–28

Harris RA, Hiles ID, Page MJ and O'Hare MJ (1995) The induction of apoptosis in human mammary luminal epithelial cells by expression of activated *c-neu* and its abrogation by glucocorticoids. *Br J Cancer* **72**: 386–392

Hockenbery D, Nunez G, Milliman C, Schreiber RD and Korsemeyer SJ (1990). Bcl-2 is an inner mitochondrial membrane protein that blocks programmed cell death. *Nature* **348**: 334–336

Hockenbery DM, Zutter M, Hickey W, Nahm M and Korsmeyer SJ (1991) Bcl-2 protein is topographically restricted in tissues characterised by apoptotic cell death. *Proc Natl Acad Sci USA* **88**: 6961–6965

Holland PA, Knox WF, Potten CS, Howell A, Anderson E, Baildam AD and Bundred NJ (1997) Assessment of hormone dependence of comedo ductal carcinoma in situ of the breast. *J Natl Cancer Inst* **89**: 1059–1065

Holland R, Hendriks JHLL, Verbeek ALM, Mravunac M and Schuurmans-Stekhoven JH (1990) Extent, distribution and mammographic/histological correlations of breast ductal carcinoma in situ. *Lancet* **335**: 519–522

Johnston SRD, Maclennan KA, Sacks NPM, Salter J, Smith IE and Dowsett M (1994) Modulation of bcl-2 and Ki67 expression in oestrogen receptor-positive human breast cancer by Tamoxifen. *Eur J Cancer* **30A**: 1663–1669

Kerr JFR, Winterford CM and Harmon BV (1994) Apoptosis. Its significance in cancer and cancer therapy. *Cancer* **73**: 2013–2126

Lagios MD (1990) Ductal carcinoma in situ. Pathology and treatment. *Surg Clin North Am* **70**: 853–871

Leek RD, Kaklamanis L, Pezzella F, Gatter K and Harris AL (1994) *bcl-2* in normal human breast and carcinoma, association with oestrogen-receptor positive, epidermal growth factor receptor-negative tumours and *in situ* cancer. *Br J Cancer* **69**: 135–139

Lipponen P, Aaltomaa S, Kosma VM and Syrjänen K (1994) Apoptosis in breast cancer as related to histopathological characteristics and prognosis. *Eur J Cancer* **30A**: 2068–2073

Merritt AJ, Potten CS, Watson AJM, Loh DY, Nakayama K, Nakayama K and Hickman JA (1995) Differential expression of bcl-2 in intestinal epithelia. Correlation with attenuation of apoptosis in colonic crypts and the incidence of colonic neoplasia. *J Cell Sci* **108**: 2261–2271

Page DL and Lagios MD (1995) Pathological analysis of the NSABP-B17 trial. *Cancer* **75**: 1219–1222

© Cancer Research Campaign 1998 

Page DL, Dupont WD, Rogers LW and Landenberger M (1982) Intraductal carcinoma if the breast: follow-up after biopsy only. *Cancer* **4**: 751–758

Page DL, Anderson TJ and Rogers LW (1989) Carcinoma in situ. In *Diagnostic Histopathology of the Breast*, Page DL, Anderson TJ (eds) pp. 43–61. Edinburgh: Churchill Livingstone

Potten CS (1992) The significance of spontaneous and induced apoptosis in the gastrointestinal tract of mice. *Cancer Metastasis Rev* **11**: 179–195

Potten CS (1996) What is apoptotic index measuring? A commentary. *Br J Cancer* **74**: 1743–1748

Potten CS, Watson RJ, Williams GT, Tickle S, Roberts SA, Harris M and Howell A (1988) The effect of age and menstrual cycle upon proliferative activity of the normal human breast. *Br J Cancer* **58**: 163–170

Sabourin JC, Martin A, Baruch J, Truc JB, Gompel A and Poitout P (1994) *bcl-2* expression in normal breast tissue during the menstrual cycle. *Int J Cancer* **59**: 1–6

Schwartzman RA and Cidlowski JA (1993) Apoptosis, the biochemistry and molecular biology of programmed cell death. *Endocr Rev* **14**(2): 133–151

Sierra A, Lloveras B, Castellsague X, Moreno L, Garcia-Ramirez M and Fabra A (1995) *Bcl-2* expression is associated with lymph node metastasis in human ductal breast carcinoma. *Int J Cancer* **69**: 54–60

Siziopikou KP, Prioleau JE, Harris JE and Schnitt SJ (1996) *bcl-2* expression in the spectrum of preinvasive breast lesions. *Cancer* **77**: 499–506

Solin LJ, Yeh IT, Kurtz J, Fourquet A, Recht A and Kuske R, McCormick B, Cross MA, Schultz DJ, Amalric R, Livolsi VA, Kowalyshyn MJ, Torhorst J, Jacquemier J, Westermann CD, Mazoujian G, Zafrani B, Rosen PP, Goodman RL and Fowble BL (1993) Ductal carcinoma in situ (intraduct carcinoma) of the breast treated with breast conserving surgery and definitive irradiation. Correlation of pathological parameters with outcome of treatment. *Cancer* **71**: 2532–2542

Symonds H, Krall L, Remington L, Saenz-Robles M, Lowe S, Jacks T and Van Dyke T (1994) p53-dependent apoptosis suppresses tumour growth and progression in vivo. *Cell* **78**: 703–711

Tsuda H, Fukutomi and Hirohashi S (1995) Pattern of gene alterations in intraductal breast neoplasms associated with histological type and grade. *Clin Cancer Res* **1**: 261–267

Van De Vijver MJ, Peterse JL, Mooi WJ, Wisman P, Lomans J, Dalesio OA and Nusse R (1988) *Neu* protein overexpression in breast cancer: association with comedo-type ductal carcinoma in situ and limited prognostic value in stage II breast cancer. *New Engl J Med* **319**: 1239–1245

Weiss LM, Warnke RA, Sklar J and Cleary ML (1987) Molecular analysis of the t(14,18) chromosomal translocation an malignant lymphomas. *New Engl J Med* **317**: 1185–1189

© Cancer Research Campaign 1998